# One or two serological assay testing strategy for diagnosis of HBV and HCV infection? The use of predictive modelling

**DOI:** 10.1186/s12879-017-2774-1

**Published:** 2017-11-01

**Authors:** John V. Parry, Philippa Easterbrook, Anita R. Sands

**Affiliations:** 1 Virus Reference Department, Public Health England, 61 Colindale Avenue, London, NW9 5HT UK; 2 Centre for Research on Drugs & Health Behaviour, London School of Hygiene & Tropical Medicine, London, UK; 3 Global Hepatitis Programme, HIV Department, World Health Organization, Geneva, Switzerland; 4 Department of Essential Medicines and Health Products, World Health Organization, Geneva, Switzerland

## Abstract

**Background:**

Initial serological testing for chronic hepatitis B virus (HBV) and hepatitis C virus (HCV) infection is conducted using either rapid diagnostic tests (RDT) or laboratory-based enzyme immunoassays (EIA)s for detection of hepatitis B surface antigen (HBsAg) or antibodies to HCV (anti-HCV), typically on serum or plasma specimens and, for certain RDTs, capillary whole blood. WHO recommends the use of standardized testing strategies – defined as a sequence of one or more assays to maximize testing accuracy while simplifying the testing process and ideally minimizing cost. Our objective was to examine the diagnostic outcomes of a one- versus two-assay serological testing strategy. These data were used to inform recommendations in the 2017 WHO Guidelines on hepatitis B and C testing.

**Methods:**

Few published studies have compared diagnostic outcomes for one-assay versus two-assay serological testing strategies for HBsAg and anti-HCV. Therefore, the principles of Bayesian statistics were used to conduct a modelling exercise to examine the outcomes of a one-assay versus two-assay testing strategy when applied to a hypothetical population of 10,000 individuals. The resulting model examined the diagnostic outcomes (true and false positive diagnoses; true and false negative diagnoses; positive and negative predictive values as a function of prevalence; and total tests required) for both one-assay and two-assay testing strategies. The performance characteristics assumed for assays used within the testing strategies were informed by WHO prequalification assessment findings and systematic reviews for diagnostic accuracy studies. Each of the presumptive testing strategies (one-assay or two-assay) was modelled at varying prevalences of HBsAg (10%, 2% and 0.4%) and of anti-HCV (40%, 10%, 2% and 0.4%), aimed at representing the range of testing populations typically encountered in WHO Member States. When the two-assay testing strategy was considered, the model assumed the independence of the two assays.

**Results:**

Modeling demonstrated that applying a single assay (HBsAg or anti-HCV), even with high specificity (99%), may result in considerable numbers of false positive diagnoses and low positive predictive values (PPV), particularly in lower prevalence settings. Even at very low prevalences shifting to a two-assay testing strategy would result in a PPV approaching 1.0. When test sensitivity is high (>99%) false negative reactions are rare at all but the highest prevalences; but a two-test strategy might yield more false negative diagnoses. The order in which the tests are used has no impact on the overall accuracy of a two-assay strategy though it may impact the total number of tests needed to complete the diagnostic strategy, incurring added cost and complexity. HBsAg assays may have a low sensitivity (<90%), and result in large numbers of false negative diagnoses, particularly in high prevalence settings, which would be exacerbated in the two-assay testing strategy. In contrast, most anti-HCV assays have high sensitivity and lead to fewer false negative results, both in the one-assay and two-assay testing strategies. At prevalences ≤2% the number of tests needed using a second assay was nearly always small, at <300 per 10,000 individuals tested, making sustainability of a second assay uncertain in such a setting.

**Conclusions:**

A key public health objective of an effective testing strategy is to identify all individuals who would benefit from treatment. Therefore, a strategy that prioritizes a high NPV (minimal false negatives) may be acceptable even if the PPV is suboptimal (some false positives) as the implementation of such a public health programme must also take account of other factors such as costs, feasibility, impact on testing uptake and linkage to care, and consequences of a false-positive test. This rationale informed the development of the WHO Viral Hepatitis Testing Guidelines, with a conditional recommendation for a one-assay serological testing strategy in most testing settings and populations (≥0.4% prevalence in population tested). A one-test strategy results in few failures to diagnose infection and, although it is associated under most assumptions with a sub-optimal PPV, benefits include greater simplicity, easier implementation, lower costs and better feasibility, uptake and linkage to care. Furthermore, prior to antiviral therapy all those diagnosed either HBsAg or anti-HCV positive will require confirmation of viræmia, preventing unnecessary treatment of those who may be false positive on serology. For HBsAg, in low-prevalence settings (≤0.4%), a second recommendation was made to consider a two-assay testing strategy, using a confirmatory neutralization step or a second different HBsAg assay.

**Electronic supplementary material:**

The online version of this article (10.1186/s12879-017-2774-1) contains supplementary material, which is available to authorized users.

## Background

Serological assays are typically used as the first line of a testing strategy for diagnosis of chronic infection with hepatitis B virus (HBV) or hepatitis C virus (HCV) infection, to rule in those who might potentially be infected and therefore benefit from assessment for treatment intitiation, and to rule out uninfected individuals. However, given that no one assay is ever 100% sensitive or 100% specific, it is likely that some uninfected individuals may be “ruled-in” as HBV or HCV infected while some infected individuals may be “ruled-out”. The objective of testing strategies is to minimize the possibility for such misdiagnoses through determining the optimal number and sequence of tests that should be conducted to ensure the positive and negative predictive values, i.e. the probability that the test result is correct is as close to 1.0 (≡100%) as possible. For HBV, the presence of hepatitis B surface antigen (HBsAg) indicates current (acute or chronic) HBV infection. For HCV, the presence of antibodies to HCV (anti-HCV) indicates that an individual has been exposed to HCV, and of these 60%–80% will currently be infected and HCV viraemic, and the remainder will have spontaneously cleared infection [1]. Both HBsAg and anti-HCV may be tested using serum or plasma specimens with laboratory-based enzyme immunoassays (EIAs). Many rapid diagnostic tests (RDTs) for both HBsAg and anti-HCV use venous or capillary whole blood, and some even oral fluid in the case of anti-HCV, providing a potential for quick results at or near to point of care. While serological assays may be associated with performance claims of high sensitivity and specificity (up to 99%), when used to test very large numbers of individuals, the number of false positive diagnoses will outnumber true diagnoses, particularly when prevalence is low [2]. The causes of false reactivity are diverse, including cross-reactions due to antibody responses to other pathogens, immunizations, autoantibodies, and errors. In such circumstances, reflexing the specimen onto a second serological assay with the intent of confirming the initially observed reactivity will improve differentiation of true positive specimens from false positive specimens prior to more expensive nucleic acid testing (NAT) technologies for direct viral detection and staging of liver disease to guide the need for treatment.

**Fig. 1:**
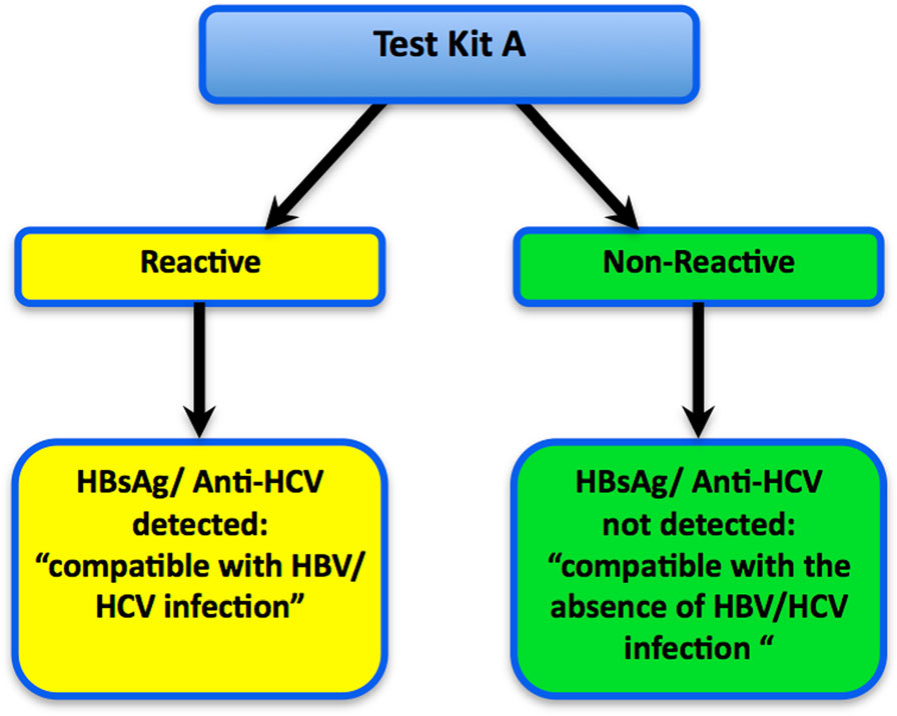
One-assay testing strategy

**Fig. 2:**
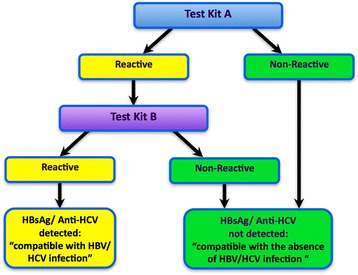
Two-assay testing strategy applying independent tests in sequence

WHO recommends the use of standardized testing strategies – i.e. the number of assays and sequence of testing to be followed to maximize the accuracy of HBsAg or anti-HCV testing while simplifying the process to minimize the number of assays (and tests on those assays) required. This was achieved with HIV diagnostic testing and led to the development of WHO guidance on standardized HIV testing strategies, first in 1992 [3] which were revised in 1997 [4]. Recommended testing strategies were based on the prevalence of HIV in the community being tested and an acceptable positive predictive value (PPV), as close to 100% as possible. The testing strategies were generic, not specifying individual assay brands or assay formats, but providing a decision flow chart and guidance about how to select a combination of distinct commercial assays available on their respective markets and validate them as an integrated and reliable testing algorithm. A one-assay testing strategy (Fig. 1) is when a diagnostic test is performed on a single serological assay. This testing strategy is particularly suitable for higher prevalence settings as the PPV will be relatively high when a highly specific assay is employed. A two-assay testing strategy (Fig. 2) employs two different assays in sequence in the expectation that this would provide a better PPV than that of a one-assay testing strategy, thus reducing the number of individuals with false positive results that are unnecessarily referred for additional testing and/or clinical assessment.

Tests to confirm serological positivity can generally be undertaken by either (i) repeating the serological test using a different commercial assay of similar sensitivity, or ii) in the case of HBsAg, performing a neutralization step using a specific anti-HBs-containing reagent. In some cases this may be available as an adjunct to the same first-line HBsAg assay. The ultimate choice between a one-assay versus two-assay serological testing strategy will depend on the seroprevalence in the population to be tested and diagnostic accuracy (sensitivity and specificity) of the assays used.

To date there have been limited data to guide the optimal selection of testing strategies in different epidemic settings for viral hepatitis. Our objective was to undertake a formal evidence review and predictive modelling to compare a one versus two serological assay testing strategy to inform the development of recommendations in the 2017 WHO viral hepatitis testing guidelines [5].

## Methods

Systematic reviews were commissioned for the development of the WHO guidance on viral hepatitis testing services, described in detail in Amini et al. [6] and Tang et al. [2]. Those reviews identified only one study that had compared the diagnostic accuracy of different testing strategies and this was only for detection of anti-HCV [7]. As the published literature was unable to provide direct evidence to answer the question of a one-assay versus a two-assay testing strategy for HBsAg and for anti-HCV respectively, a model was developed using the principles of Bayesian statistics. The diagnostic accuracy of a one-assay or two-assay testing strategy in a hypothetical population of 10,000 individuals was modelled across a range of prevalences 40% (HCV only); 10%; 2%; 0.4% representing typical testing situations. Similarly, a range of performance characteristics for assays was employed to model the minimum sensitivity and specificity that would be expected for assays chosen for use within the testing strategies. These performance characteristics were taken from WHO prequalification assessments and other systematic reviews commissioned for the development of the WHO guidance on viral hepatitis testing services [2, 6, 8].

For HBsAg, four combinations of assay sensitivity and specificity were modelled in a one-assay testing strategy (Table 1a) representing typical assay performance observed in WHO prequalification assessment (test kits 1, 3) and in the published studies from settings that are typical of the intended use (test kits 2, 4). Two models based on two-assay testing strategies were also evaluated, one reflecting a combination of assay performance typical of intended use setting study findings (test kits 2 + 5), the other reflecting assay performance from the WHO prequalification assessment studies (test kits 1 + 6).

**Table 1:** a. HBsAg: Assay sensitivities and specificities employed in models for 4 representative test kits (1–4) used in a modelled 1-test strategies and 2 pairs (Kits 2 + 5; 1 + 6) in 2-test strategies. 1b. Anti-HCV: Assay sensitivities and specificities employed in models for 4 representative test kits (1–4) used in modelled 1-test strategies and 2 pairs (Kits 2 + 5; 3 + 4) in 2-test strategies

1a.	1-Test Kit Strategies				2-Test Kit Strategies			
Assay A	Kit 1	Kit 2	Kit 3	Kit 4	Kit 2	**↘**	Kit 1	**↘**
Assay B	–	–	–	–		Kit 5		Kit 6
Sensitivity	98%	90%	98%	80%	90%	90%	98%	98%
Specificity	99%	99%	98%	99%	99%	99%	99%	99%
1b.	1-Test Kit Strategies				2-Test Kit Strategies			
Assay A	Kit 1	Kit 2	Kit 3	Kit 4	Kit 2	**↘**	Kit 3	**↘**
Assay B	–	–	–	–		Kit 5		Kit 4
Sensitivity	99%	98%	99.5%	85%	98%	98%	99.5%	85%
Specificity	99%	99%	98%	99%	99%	98%	98%	99%

For the anti-HCV testing strategy, four combinations of assay sensitivity and specificity were modelled in a one-assay testing strategy (Table 1b). Unlike HBsAg assays, typical anti-HCV assay performance observed in WHO prequalification assessment were similar to the published field studies, both sources showing very high sensitivity and specificity, though one study did report lower sensitivity and this is reflected in the parameters used for Kit 4. Of the two-testing strategies modeled for anti-HCV, one reflected a combination of kits typical of the pooled intended use setting studies and WHO prequalification assessment studies (Kits 2 + 5), the other incorporating a highly sensitive and specific first-line assay (Assay A: Kit 3) together with a second-line assay with poor sensitivity (Assay B: Kit 4).

### Testing strategies investigated

Figures 1 and 2 summarize the one-assay and two-assay testing strategies modelled. With the one-assay testing strategy, if the test result is reactive, a status “compatible with presence of HBV/ HCV infection” is reported with onward referral for further testing and clinical investigations. If the initial test result is non-reactive, a status “compatible with the absence of HBV/HCV infection” is reported.

A two-assay testing strategy differs in that two different assays are used sequentially, such that the second assay is applied only if the first test result is reactive. The aim of such a strategy is to improve the PPV of the testing, and so reduce the number of individuals inappropriately referred on to more specialist testing services. Consequently, if either test result is non-reactive, a status of “compatible with the absence of HBV/HCV infection” would be reported. If both test results are reactive, the status would be reported as “presumptive HBV/HCV infection for further diagnostic testing”. The option to incorporate a third possible outcome of an “inconclusive” status into a two-assay testing strategy was rejected primarily because discrepant testing results are most frequently associated with false reactivity in the first-line assay. Using the results of a third-line assay in a tie breaker fashion (where a reactive result for the third assay would rule in the status as ‘positive’) would lead to substantial reduction in the PPV of the strategy, increased cost, and/ or many uninfected individuals being unnecessarily referred for more complex testing and/or clinical evaluation at additional expense both to the individual and to health services [9].

### Data sources for prevalence and diagnostic performance in predictive model

#### Diagnostic performance

A series of assay sensitivities and specificities was used in the models that were based on pooled estimates from two recent systematic reviews on diagnostic performance of RDTs and EIAs for detection of HBsAg and anti-HCV commissioned to inform the WHO viral hepatitis testing guidelines, as well as those used in performance evaluations conducted as part of WHO prequalification assessment [2, 6, 8].

##### Systematic reviews

**HBsAg:** Based on a systematic review of 30 studies [6], the pooled clinical sensitivity of HBsAg RDTs against different EIA as reference standards was 90.0% (95% CI: 89.1–90.8) and pooled specificity was 99.5% (95% CI 99.4–99.5). There was significant variation in performance between studies, RDT brands and within the same brand of RDT, with sensitivity ranging from 50% to 100% (and for EIA 74%–100%) and specificity from 69% to 100%.

**Anti-HCV:** Based on a systematic review of 52 studies [2], the pooled clinical sensitivity and specificity of anti-HCV RDTs were, respectively, 99% (95% CI: 98–100) and 100% (95% CI: 100–100), but sensitivities in individual studies ranged from 83% to 100%, and specificities from 99% to 100%. There was significant heterogeneity between studies and variable performance across RDT brands and even within the same brand.

A key limitation of these studies was their use of a single EIA result (generally the assay in routine use by the reporting laboratory) as a reference standard and without further serological confirmation, which is a source of uncertainty with respect to the performance estimates.

##### WHO prequalification data

The other performance data used in the models were those generated through performance evaluations conducted for WHO prequalification assessment, whose specimen panels are fully characterized and the specimens collected from worldwide sources [8]. Tables 1a and b show the four combinations of sensitivity and specificity employed in the modelled one-assay and two assay testing strategies for HBsAg and anti-HCV, respectively.

### HBsAg and anti-HCV population seroprevalence

Three HBsAg seroprevalence levels (10%, 2% 0.4%) and four anti-HCV seroprevalence levels (40%, 10%, 2% 0.4%) representing typical very high- (for anti-HCV), high-, medium- and low-seroprevalence settings were utilized in the models. These were derived from a series of published systematic reviews of prevalence of anti-HCV and of HBsAg in different populations [10].

**For HBsAg,** a prevalence of 10% was used to represent high endemicity regions, where mother to child transmission is the main transmission route for chronic hepatitis B infection such as Sub-Saharan Africa; 2% to represent settings in which HBV infection is common, such as PWID, sex workers and MSM in concentrated epidemic settings, and 0.4% to reflect low prevalence settings or populations [1, 10].

**For Anti-HCV:** a prevalence of 40% was used to represent high prevalence populations, e.g. PWID, or settings, e.g. drug treatment programmes or prisons [11]; 10% to represent regions in which HCV has become well established among the general population, such as in parts of North Africa, Middle East and Central and East Asia; 2% to represent regions such as parts of Europe, Australasia, South/ South-East Asia and Sub-Saharan Africa; and 0.4% to reflect low general population prevalence settings outside of high-risk groups in North America, and tropical Latin America [1].

## Modelling calculations

Table 2 shows the formulae applied to estimate the overall diagnostic performance for a one-assay (Fig. 1) and two-assay (Fig. 2) testing strategy. To facilitate understanding of the impact, these parameters were applied to a model population of 10,000 individuals to generate expected numbers of each diagnostic outcome: true positive; false positive; true negative; false negative. In addition, PPV, NPV and ratio of true positive:false positive diagnoses were determined for each representative prevalence and testing strategy. For the two-assay testing strategy, the number of tests to be performed on the second assay was also calculated as this has a bearing on the costs and sustainability of having a second assay available in testing sites. For the purposes of the two-assay testing strategy, the assumption was made that the performance characteristics (sensitivity and specificity) of assay B were not influenced by the process of sub-selecting initially reactive specimens to be tested on assay B. In practice, however, it is likely that selection by assay A of reactive specimens to be tested in assay B will affect the sensitivity and specificity claimed by an assay manufacturer that has been determined without such selective pressure, and this is discussed in greater detail below.

**Table 2:** Formulæ employed to calculate outcomes of applying a one- or two-test diagnostic strategy to a population of 10,000 individuals with several prevalences of infections

One-Test Strategy		Two-Test Strategy	
TP1=	N x E x SenA	TP2 =	TP1 x SenB
TN1 =	(N x (1 – E)) – (N x (1 – E) x (1 – SpecA))	TN2 =	TN1 + (FP1 x SpecB)
FP1 =	N x (1 – E) x (1 – SpecA)	FP2 =	FP1 x (1 – SpecB)
FN1 =	N x E x (1 – SenA)	FN2 =	FN1 + (TP1 x (1 – SenB))
PPV1 =	TP1 / (TP1 + FP1)	PPV2 =	TP2 / (TP2 + FP2)
NPV1 =	TN1 / (TN1 + FN1)	NPV2 =	TN2 / (TN2 + FN2)
POR1 =	TP1 / FP1	POR2 =	TP2 / FP2

*N* Population size, *E* Prevalence of infection, *TP* True positive, *SenA* Assay A sensitivity, *SpecA* Assay A specificity, *TN* True negatives, *SenB* Assay B sensitivity, *SpecB* Assay B specificity, *FP* False positives, *PPV* Positive predictive value, *FN* False negatives, *NPV* Negative predictive value, *POR* Ratio of true to false positive tests

The two assays were assumed to be fundamentally distinct from each other, such that the sensitivity performance claims for assay B were used to calculate the probability that an individual who has the infection and has been found reactive by assay A would also be found reactive in assay B. Equally, specificity claims for assay B were used to calculate the probability that an individual for whom a false positive reaction arose in assay A would be non-reactive in assay B. When these were applied to a population of defined size, e.g. 10,000, the numbers of true positive, false positive, true negative and false negative diagnoses were predicted.

## Results

### Relationship between testing strategy, prevalence of infection, assay sensitivity and specificity, and PPV and NPV

#### One-assay testing strategy

##### Impact of prevalence

**Fig. 3:**
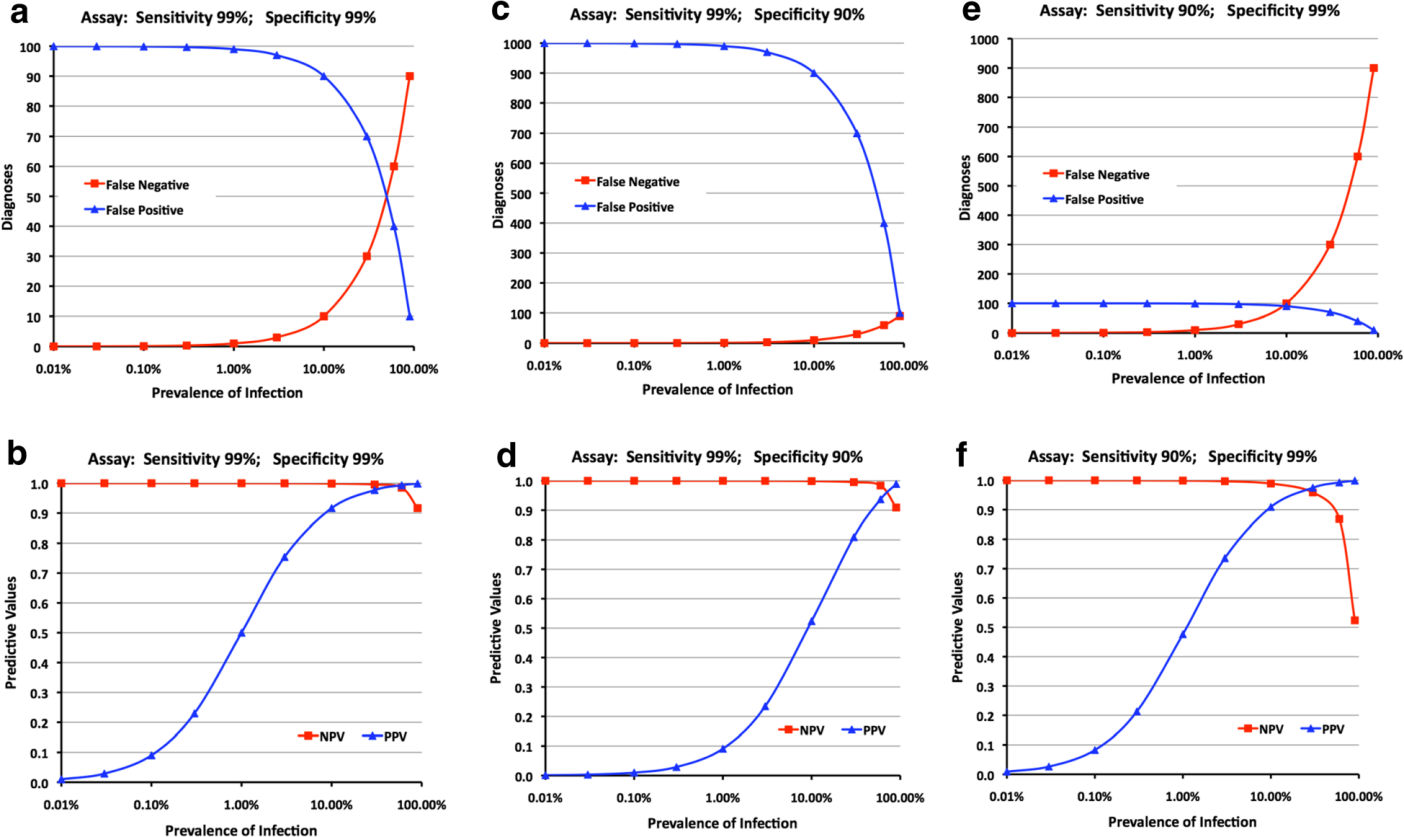
**a**-**f**: Employing a one-test strategy (Fig. 1), relationships between prevalence of infection, assay sensitivity and specificity, false reactions and positive and negative predictive values

When applying a one-assay testing strategy, even assays with a specificity as high as 99%, such as in the worked example in Fig. 3a-b, gave rise to considerable numbers of false positive results, and a poor PPV at all but the highest of prevalences. At a prevalence of 1%, false positive results equaled true positive results (PPV 0.5), and at prevalences of <0.1%, false positive diagnoses outnumbered true positive diagnoses by >10-fold. Only when the prevalence of anti-HCV or HBsAg increased (i.e. fewer people without anti-HCV or HBsAg in the testing population), did PPV increase such that when prevalence reached almost 10%, true positive results outweighed false positive results by 10-fold.

##### Impact of assay performance

Reducing assay specificity to 90% increased the number of false positive results 10-fold, from 90-100 to 900–1000 at prevalences <10%, and the PPV decreased to 0.09 at a 1% prevalence (Fig. 3c-d). At a 10% prevalence, PPV had risen to just over 0.5 which means, for example, when HBsAg prevalence was 10%, there is only a 50% probability that a person with a positive HBsAg result was truly positive for HBsAg. A more optimal PPV >0.9 was not reached unless prevalence was ≥50%, which would rarely happen, especially for HBsAg.

Reducing assay sensitivity to 90% had no impact on the number of false positives and PPV (Fig. 3e). When assay sensitivity was high (99%), false negative results were rare for all but the highest prevalences (Fig. 3a; c) as specimens from individuals whose specimen containing HBsAg or anti-HCV were scarce. Even at 10% prevalence, only 10 false negative results arose among the 10,000 tests done, and the NPV was 0.999. It was only at very high prevalence, e.g. ≥30%, that any impact on NPV became discernable (Fig. 3b; d), as it fell to 0.985 and 0.917 at prevalences of 60% and 90% respectively. Reducing sensitivity to 90% increased the number of false negative results 10-fold (Fig. 3e), with concomitant substantial falls in NPV at higher prevalences, falling to 0.959, 0.868 and 0.524 at prevalence of 30%, 60% and 90% respectively (Fig. 3f).

#### Two-assay testing strategy

The serial two-assay testing strategy, illustrated in Fig.2, employed a second assay (assay B) to test all specimens reactive in the first-line assay (assay A).

##### Impact of prevalence

**Fig. 4:**
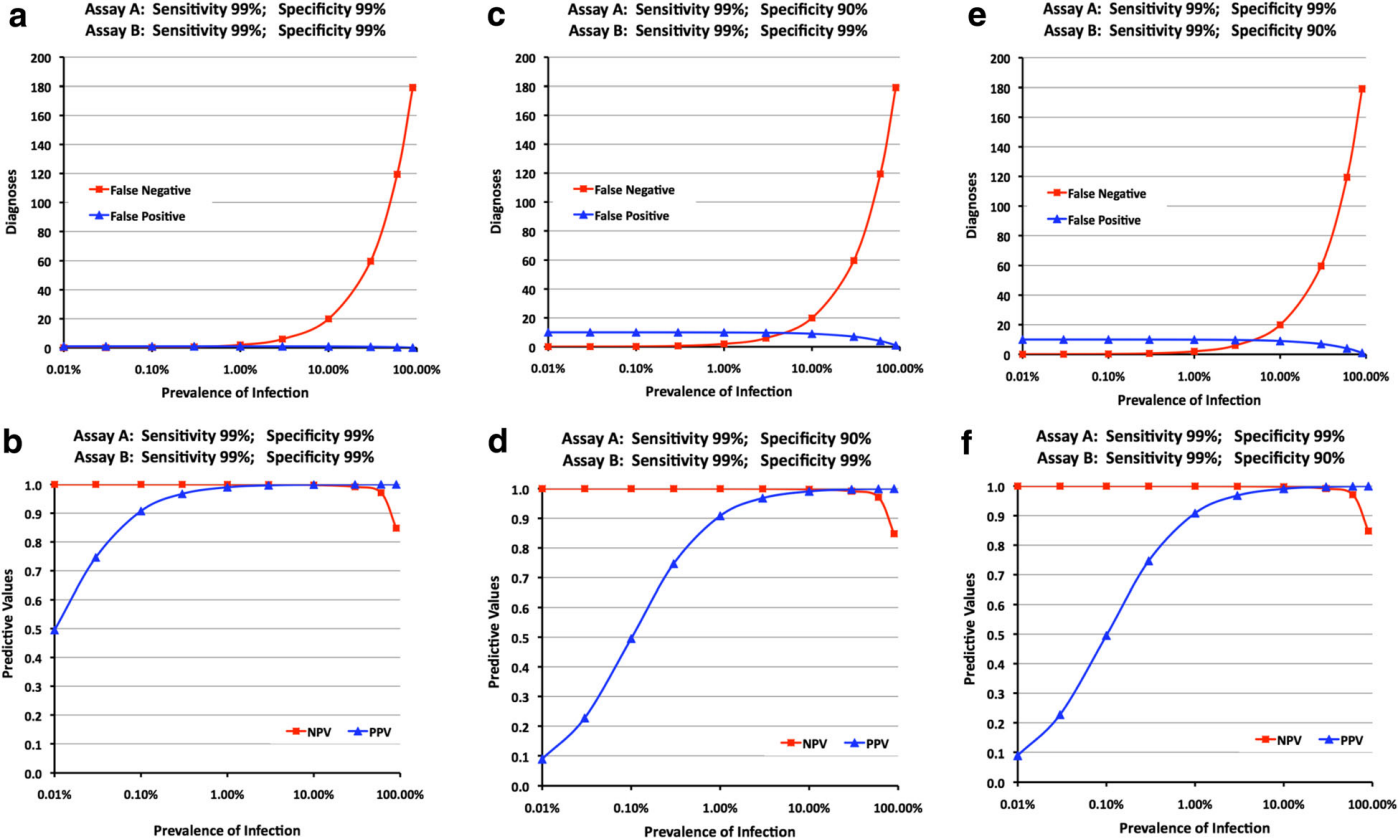
**a**-**f**: Employing a two-test strategy (Fig. 2), relationships between prevalence of infection, assay sensitivity and specificity, false reactions and positive and negative predictive values

Even at the lowest prevalence, the introduction of the second assay reduced the total number of false positive diagnoses, making them very rare, thus increasing the PPV (Fig. 4a-f) compared to the equivalent single assay testing strategy.

##### Impact of assay performance

Employing assays that were both sensitive (99%) and specific (99%) meant that false positive diagnoses were quite rarely observed, even at the lowest prevalence, with PPVs >0.99 at any prevalence >1% (Fig. 4a; b). When an assay A with reduced specificity (90%) was followed by assay B of 99% specificity, greatly reduced numbers of false positive diagnoses were observed compared with the one-assay testing strategy, even at prevalences below 1%, showing reductions from 1000 to 10 (Fig. 4c cf. Figure 3c). PPV was also substantially improved to ~0.5 and ~0.9 at prevalence of 0.1% and 1% respectively as opposed to 0.1 and 0.5 for the one-assay testing strategy (Fig. 4d cf. Figure 3d).

However, comparing the one-assay with the two-assay testing strategy, the models yielded at each prevalence double the number of false negative diagnoses, though this is not apparent until the prevalence approaches 1% and above, due to the associated rarity of infected individuals. Reduced specificity had no impact on this (Fig. 4a cf. Figure 4c). Similarly, NPV was at, or close to, 1.0 until prevalence rose to 30% and above (Figs. 4b, d).

##### Impact of sequence of assays

**Fig. 5:**
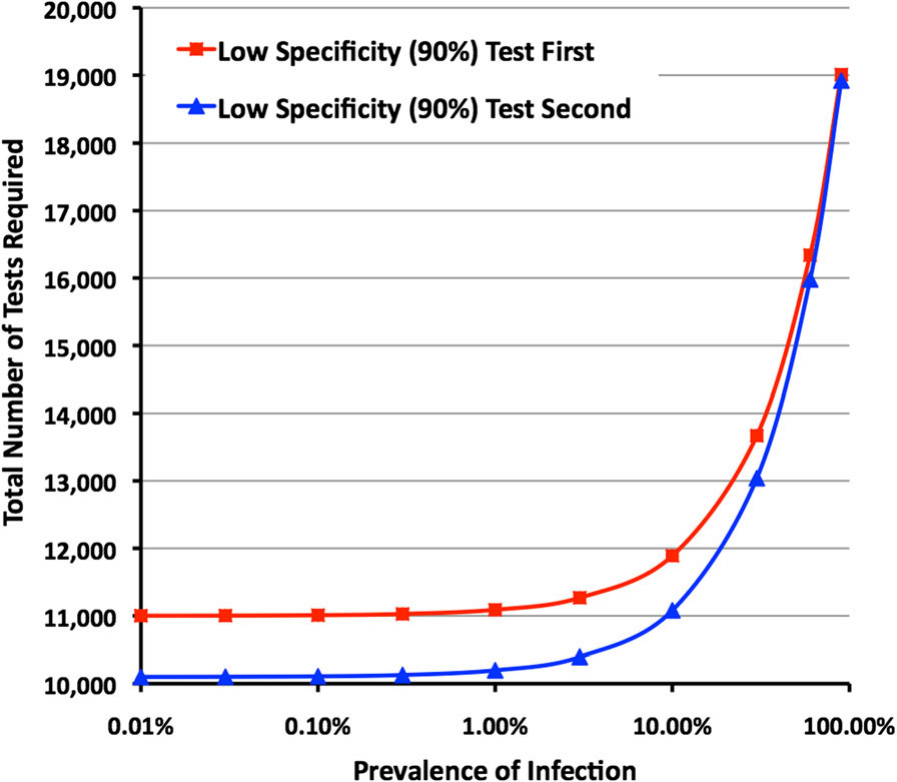
Impact of the order in which test kits are applied and prevalence of infection on the total number of tests needed

We also explored whether the sequence in which Assay A and Assay B were applied had any impact on the four diagnostic categories. We reversed the order in which the assays were applied to the model such that a highly sensitive (99%) and specific (99%) assay was employed first, followed by an assay with reduced specificity (90%). The numbers of specimens in each diagnostic category and associated predictive values were unchanged (Figs. 4c, d cf. Figure 4e, f), indicating that the order has no impact on the overall accuracy of a two-assay testing strategy that follows the pattern illustrated in Fig. 2. However, the order in which assays were applied in a two-assay testing strategy does have an impact on the total number of tests needed to complete the testing strategy for the model population of 10,000. At prevalences of up to 3%–5% an additional 700–1000 tests were needed when the lower specificity assay was employed first, the difference gradually diminishing as the prevalence was increased to levels above 50% (Fig. 5).

### Disease-specific findings

#### Hepatitis B virus - HBsAg

The outcomes from applying one-assay and two-assay testing strategy models to three scenarios in which populations of 10,000 individuals with respective HBsAg prevalences of 10%, 2% and 0.4% are given in Additional file 1. For each scenario several test kits (test kits 1-6), with hypothetical performance characteristics (Table 1a) were applied either singly (test kits 1-4) or in combinations of two (test kits 1 + 5; 2 + 6) according to the testing strategies described in Figs. 1 and 2. An overview of the findings has been extracted into Table 3, contrasting one-assay with two-assay testing strategies and comparing WHO prequalified test kits (e.g. test kits 1 & 6) with test kits in intended use studies (e.g. test kits 2 & 5), whose pooled sensitivity was much reduced, at ~90%.

**Table 3:** Selected outcomes from applying 1- and 2-test diagnostic strategy models to three HBV epidemic scenarios. The examples here have been extracted from Additional file 1

Population: 10,000							
	Strategy	HBsAg Test Kit	True Positive	False Positive	False Negative	PPV	number of assay B tests
Scenario 1 Prevalence 10%:	1-test	1	980	90	20	0.916	
	2-test	1 → 6	960	1	40	0.999	1070
	1-test	2	900	90	100	0.909	
	2-test	2 → 5	810	1	190	0.999	990
Scenario 2 Prevalence 2%:	1-test	1	196	98	4	0.667	
	2-test	1 → 6	192	1	8	0.995	294
	1-test	2	180	98	20	0.647	
	2-test	2 → 5	162	1	38	0.994	278
Scenario 3 Prevalence 0.4%	1-test	1	39	100	1	0.282	
	2-test	1 → 6	38	1	2	0.975	139
	1-test	2	36	100	4	0.265	
	2-test	2 → 5	32	1	8	0.970	136
Test Kit Performance Characteristics:							
Test Kits	Sensitivity	Specificity					
1 and 6	98.0%	99.0%					
2 and 5	90.0%	99.0%					
Notes on Two-Test Strategies:							
Assay B performance is considered independent of Assay A							
Outcome of 2-test strategy: A + B+ = pos and A-, A + B- = neg (Fig. 2)							
2-test strategies overall performance:				Assay A	Assay B	Sensitivity	Specificity
				1	6	96.04%	99.99%
				2	5	81.00%	99.99%

##### Specificity

The most remarkable finding was the vast improvement in specificity afforded by applying a two-assay testing strategy, the 90–100 false positive results arising in the first-line assay was reduced in all cases to a hypothetical single false positive diagnosis. Allied to this was a substantial increase in PPV, particularly marked at lower prevalences.

##### Sensitivity

Unlike specificity, which was identical both in WHO prequalified test kits (e.g. test kit 1 & 6) and test kits used in the intended use setting studies (e.g. test kits 2 & 5), sensitivity was inferior in the latter, yielding 4–5 times more false negative diagnoses in the one-assay testing strategy. The model suggests that false negative diagnoses might increase up to 2-fold when applying a two-assay testing strategy such as that used in Scenario 1 (10% prevalence). Indeed 190 false negative results arose when applying test kits 2 and 5, representing nearly 20% of those with HBV infection, as opposed to 40 false negative results for test kits 1 and 6 (Table 3). At lower prevalences, far fewer false negative diagnoses were observed, but they would still represent a similar proportion of the infected individuals in the test population.

##### Number of tests

There was little difference between the two exemplar two-assay testing strategies examined (1➔6; 2➔5) in the number of Assay B tests needed to complete each strategy as this was primarily driven by population prevalence and sensitivity of the test kit used as Assay A, specificities being identical. In this example (2➔5) poor sensitivity in Kit 2 (90%) contributed to modestly reduced numbers as 10% of infected individuals would have been non-reactive and thus not been tested by Kit 5. For both assay combinations, at lower prevalences very few individuals would have been tested by Assay B (at 0.4%: 139 or 136/10,000).

#### Hepatitis C virus – Anti-HCV antibodies

The outcomes from applying one-assay and two-assay testing strategy models to four scenarios in which populations of 10,000 individuals with respective anti-HCV prevalence of 40%, 10%, 2% and 0.4% are given in Additional file 2. For each scenario, several test kits (test kits 1-5), with hypothetical performance characteristics (Table 1b) were applied either singly (test kits 1-4) or in combinations of two (test kits 2 + 4; 3 + 5) according to the strategies described in Figs. 1 and 2. An overview of findings has been extracted into Table 4, contrasting one-assay with two-assay testing strategies and comparing WHO prequalified test kits (e.g. test kits 2, 3 & 5) with one whose sensitivity was inferior, at ~85% (e.g. test kit 4), as observed in one of the intended use studies. This was included to illustrate the critical impact of the use of a defective product.

**Table 4:** Selected outcomes from applying 1- and 2-test diagnostic strategy models to four HCV epidemic scenarios. The examples here have been extracted from Additional file 2

Population: 10,000							
	Strategy	Anti-HCV Test Kit	True Positive	False Positive	False Negative	PPV	number of assay B tests
Scenario 1 Prevalence 40%:	1-test	2	3920	60	80	0.985	
	2-test	2 → 5	3842	1	158	1.000	3980
	1-test	3	3980	120	20	0.971	
	2-test	3 → 4	3383	1	617	1.000	4100
Scenario 2 Prevalence 10%:	1-test	2	980	90	20	0.916	
	2-test	2 → 5	960	2	40	0.998	1070
	1-test	3	995	180	5	0.847	
	2-test	3 → 4	846	2	154	0.998	1175
Scenario 3 Prevalence 2%:	1-test	2	196	98	4	0.667	
	2-test	2 → 5	192	2	8	0.990	294
	1-test	3	199	196	1	0.504	
	2-test	3 → 4	169	2	31	0.989	395
Scenario 4 Prevalence 0.4%	1-test	2	39	100	1	0.282	
	2-test	2 → 5	38	2	2	0.951	139
	1-test	3	40	199	0	0.167	
	2-test	3 → 4	34	2	6	0.944	239
Test Kit Performance Characteristics:							
Test Kits	Sensitivity	Specificity		Test Kits	Sensitivity	Specificity	
2	98.0%	99.0%		4	85.0%	99.0%	
3	99.5%	98.0%		5	98.0%	98.0%	
Notes on Two-Test Strategies:							
Assay B performance is considered independent of Assay A							
Outcome of 2-test strategy: A + B+ = pos and A-, A + B- = neg (Fig. 2)							
2-test strategies overall performance:				Assay A	Assay B	Sensitivity	Specificity
				2	5	96.04%	99.98%
				3	4	84.58%	99.98%

##### Specificity

The most notable finding was the vast improvement in specificity afforded by applying a two-assay testing strategy, the 60–200 false positive results arising in Assay A, dependent on particular assay specificity and population prevalence, were reduced in all cases to a hypothetical one or two false positive diagnoses. At a 0.4% prevalence, this reversed a 1:5 ratio of true: false positive diagnoses when applying test kit 3 alone to 17:1 when test kit 4 was added as Assay B in a two-assay testing strategy. Substantial increases in PPV were also observed, particularly marked at lower prevalences.

##### Sensitivity

Employing a one-assay testing strategy, a relatively small difference in sensitivity between the two Assay A test kits used (test kit 2: 98%; test kit 3: 99.5%) yields a 4-fold difference in the number of false negative diagnoses. At a prevalence of 40%, absolute numbers of missed diagnoses are of some concern, at 80 and 20 (Table 4). At lower prevalence, ≤2%, numbers were small (0–4) when considered that this is among 10,000 tests performed. Theoretically, when employing two high sensitivity test kits in a two-assay testing strategy, e.g. test kit 2 ➔ test kit 5, false negative diagnoses might increase up to 2-fold, from 80 to 158, such as in Scenario 1 (40% prevalence); similar proportions apply to lower prevalence (Scenarios 2–4), but the numbers of false negative diagnoses were small. If test kits 3 and 4 are used together (test kit 4 sensitivity: 85%) it was observed that the capability of a two-assay testing strategy to detect anti-HCV positive individuals would be severely impaired. At higher prevalences, many true positive results detected in test kit 3 would be found negative on test kit 4 which, according to the strategy illustrated in Fig. 2, would be considered to be consistent with absence of anti-HCV antibody. While this combination would be expected in a high prevalence (40%) setting to yield only a single false positive diagnosis, there would be >600 false negative diagnoses (Table 4), reducing proportionately with reduced prevalence.

##### Number of tests

There was little difference between the two exemplar two-assay testing strategies examined (2➔5; 3➔4) in the number of second-line tests needed to complete each strategy (Table 4). Unlike for HBsAg, this was driven not only by population prevalence and the differential in sensitivity of the kits used as assay A (test kit 2: 98%; test kit 3: 99.5%), but also by the difference in specificity (test kit 2: 99%; test kit 3: 98%). Test kit 3 generated both more true positive and false positive results than test kit 2. While at a prevalence of 40% at least 4000 tests using assay B (test kit 3 or 4) would be needed, at lower prevalences few individuals would have been tested by assay B (at 0.4%: 139 or 239/10,000).

## Discussion

There were three key findings from this predictive modelling using Bayesian statistics to determine which approach is optimal to test for viral hepatitis - a one-assay or a two-assay serological testing strategy. Firstly, the modelling indicated that even at low prevalence a one-assay testing strategy efficiently identifies all but a very few individuals likely to be infected, and similarly excludes nearly all uninfected individuals. Nonetheless, as the modelling demonstrates, employing a single assay with a specificity of 99% would generate more false positive diagnoses than true ones in low prevalence settings (up to 1%). While the PPV of a one-assay testing strategy may be low, this testing strategy may be more acceptable than a two-assay testing strategy from a public health approach. The testing workflow would be less complex, the ability of testing providers to maintain proficiency in the test procedure would be maintained, procurement and supply chain management of test kits and other supplies would be easier and costs would be less. Another part of the trade-off is that any individuals identified as anti-HCV or HBsAg positive by the testing strategy would in any case need supplementary testing for HCV RNA or HCV core antigen to confirm viræmic HCV infection, or retesting for HBsAg or supplementary testing for HBV DNA to establish the need for antiviral treatment, and these would be unlikely to be available at the primary testing sites.

Secondly, the primary purpose of applying a second assay within a sequential two-assay testing strategy would be for better discrimination of true positive from false positive results, which would thereby enhance the PPV of the testing strategy. Applying two assays with specificities of ≥99% is potentially highly effective as the PPV would approach 1.0 even with a prevalence as low as 1%. Moreover, at a 0.1% prevalence, the model for the two-assay testing strategy predicted only one false positive diagnosis for every ten true positive diagnoses. Even when one of the assays employed in the testing strategy has relatively poor specificity (90%), as long as the other is highly specific, a PPV of 0.5 is achieved at a prevalence 10 times lower than that achieved by a one-assay testing strategy with an assay with 99% specificity. However, should Assay B have less than perfect sensitivity there is the potential to increase the number of false negative diagnoses over and above those arising from imperfect sensitivity in Assay A. This means that the preference between false positive and false negative results must be considered. In most circumstances, these testing strategies are used to identify individuals who are likely to be chronically infected and therefore would benefit from antiviral treatment and/or care. Therefore, these serology testing results alone would not be used to make a decision on treatment initiation meaning that poor specificity would be mitigated by the supplemental testing for HCV RNA, HCV core antigen or HBV DNA.

Thirdly, when the sequence in which two assays are used in the two-assay testing strategy modelled was compared, it was clear that the order made no difference to the diagnostic outcomes. Whether the most sensitive/ specific or least sensitive/ specific assay was used first, the same numbers of correct and incorrect diagnoses arose. If, however, one applied a non-binary approach to the two-assay testing strategy, and incorporated a third, ‘inconclusive’, category to those with discrepant test results to whom further serology testing would be offered, then clearly it would be crucial that the most sensitive assay be applied first. On the other hand, the order did impact the total number of tests needed to complete the testing strategy, fewer being needed if the more specific assay was applied first. While assay-specific costs would need also to be considered, the findings suggest that it may be more cost-effective to apply the more specific assay first in the two-assay testing strategy described here.

From a public health approach, the model and examination of programmatic preferences indicates that a one-assay testing strategy will be suitable for the majority of testing settings for both anti-HCV and HBsAg. When testing in populations with HBsAg seroprevalence of <0.4%, confirmation of HBsAg positivity on the same immunoassay with a neutralization step or a second different RDT assay for detection of HBsAg would be ideal.

Additional reasons for the one-assay, rather than a two-assay, testing strategy recommendation that was made in the WHO testing guidelines (Table 5) were as follows:

- ➢ **Costs:** There are concerns about the cost implications and feasibility of implementing a second assay, particularly at point of care and in resource-limited settings. A second different assay would need to be in stock which, particularly in low prevalence settings, would rarely be used, and an additional specimen would need to be drawn for the test on the second assay. For HCV, prioritizing confirmation of HCV viræmia in those reactive on a single anti-HCV assay may likely be more cost- and time-efficient than performing a second serological assay.
- ➢ **More rapid reporting of results:** One-assay testing strategy facilitates same day results, and will help improve access and linkage to care and clinical services, including assessment of treatment eligibility (HCV RNA or HBV DNA, viral load and liver disease staging)
- ➢ Unlike HIV there has been limited evaluation of the added value of a second assay, posing the risk that the hypothetical improvements in specificity projected by the model may not be fully realized.
- ➢ Although commercial neutralization reagents are available as supplementary components of some EIA and random access analysers to confirm the presence of HBsAg, and widely used in high income countries, there is at present no equivalent neutralisation test available in RDT format, ruling out this approach in point-of-care settings.
- ➢ A single assay provides an opportunity to offer rapid HBV vaccination as appropriate to those who are HBsAg negative, and to partners and family members of those who are positive, together with prompt advice on other measures to reduce onward transmission.
- ➢ There are minimal negative consequences from a false positive result based on a single assay because all HBsAg and anti-HCV positive patients require further evaluation with HBV DNA and HCV RNA measurement and staging of liver disease to assess eligibility for antiviral treatment. Therefore, unlike HIV, no patient would be initiated on lifelong anti-viral therapy on the basis of a single serological test result alone.
- ➢ It is common practice in many settings to perform a second test for HBsAg after six months to confirm a diagnosis of chronic HBV infection to distinguish it from acute HBV infection.
- ➢ There was a conditional recommendation to consider confirmation with a neutralization step or a second different RDT for HBsAg in very low-prevalence settings (<0.4%) to improve the PPV. This would minimise unnecessary expenditure on expensive virological testing for those with false positive diagnoses based on a single assay.

**Table 5:** WHO recommendations for viral hepatitis testing [5]

Testing strategies for diagnosis of chronic HBV infection - In settings or populations with an HBsAg seroprevalence ≥0.4%, a single serological assay for detection of HBsAg is recommended, prior to further evaluation for HBV DNA and staging of liver disease. - In settings or populations with an HBsAg seroprevalence <0.4% confirmation of HBsAg positivity on the same immunoassay with a neutralization step or a second different assay for detection of HBsAg may be considered.
Testing strategy for detection of antibodies to HCV - In adults and children older than 18 months, a single serological assay for initial detection of exposure to hepatitis C is recommended prior to supplementary nucleic acid testing (NAT) or HCV core antigen for evidence of current chronic HCV infection.

There are several caveats to the interpretation of the findings from predictive modelling as well as additional considerations associated with the use of multi-assay testing strategies. First, performance claims are often generated in ideal laboratory conditions, using highly trained staff, are likely not to be realized in field conditions, and false positive and false negative findings might arise more frequently than manufacturers’ performance claims indicate [12]. Second, diagnostic accuracy depends crucially on the choice of the second test kit employed in a local algorithm. When using a multi-test strategy samples selected for testing in test kit B have been highly selected by the application of test kit A. Consequently, performance claims made by the manufacturer of test kit B, or described in an independent evaluation, are unlikely to apply. Third, “different” RDTs may fundamentally be the same, and therefore prone to similar inaccuracies and false-positive results, e.g. different assays from the same manufacturer may use common components, or different manufacturers may source the same raw materials, or even the entire device, from the same third party supplier. In such cases, there is a greatly increased likelihood that individuals whose specimen is falsely reactive in the first assay will also be reactive in the second assay, thus eliminating any value of applying a second assay. Most critically, the translation of the testing strategy, which is generic, into a local testing algorithm employing specific test kits, needs carefully to be validated before being incorporated into routine clinical use.

In view of the scarcity of published evidence on test kit performance and testing strategies for HBV and HCV, diagnosis in resource-poor settings may be reviewed using this model based on Bayesian statistics and experience from similar approaches to HIV testing. For both HBV and HCV, there is a need for further evaluation of the diagnostic performance, impact, cost and cost–effectiveness of one- versus two-serological assay testing strategies in diverse settings of both high and low HBsAg and anti-HCV prevalence.

## Conclusion

Comparison of one-assay versus two-assay serological testing strategies for either HBsAg or anti-HCV required a more theoretical approach for evidence to support one testing strategy over the other. In this study, Bayesian statistics were used to model the two possibilities in a theoretical population of 10,000. The prevalence of HBsAg or anti-HCV in this population was varied to represent the different types of testing settings in WHO Member States. Finally, the performance characteristics of the assays used within the testing strategies were varied to mirror field realities. This model provided an objective description of the various diagnostic outcomes (e.g. sensitivity, specificity, etc.) for each of the two candidate testing strategies. This was used by the WHO Hepatitis Testing Guidelines Development Group in their deliberations of the relative trade-offs of each candidate testing strategy – the age-old debate of specificity versus sensitivity as a function of a simplified diagnostic pathway to lifesaving antiviral treatment.

## Additional files

Additional file 1: Outcomes from applying 1- and 2-test diagnostic strategy models to three HBV epidemic scenarios. (DOCX 979 kb)

Additional file 2: Outcomes from applying 1- and 2-test diagnostic strategy models to four HCV epidemic scenarios (DOCX 953 kb)
