# Supplementary material for: One or two serological assay testing strategy for diagnosis of HBV and HCV infection? The use of predictive modelling
Source: BMC Infect Dis. 2017 Nov 1;17(Suppl 1):705. doi: 10.1186/s12879-017-2774-1 (PMC5688456; doi:10.1186/s12879-017-2774-1)

**Additional file 1:** Outcomes from applying 1- and 2-test diagnostic strategy models to three HBV epidemic scenarios.


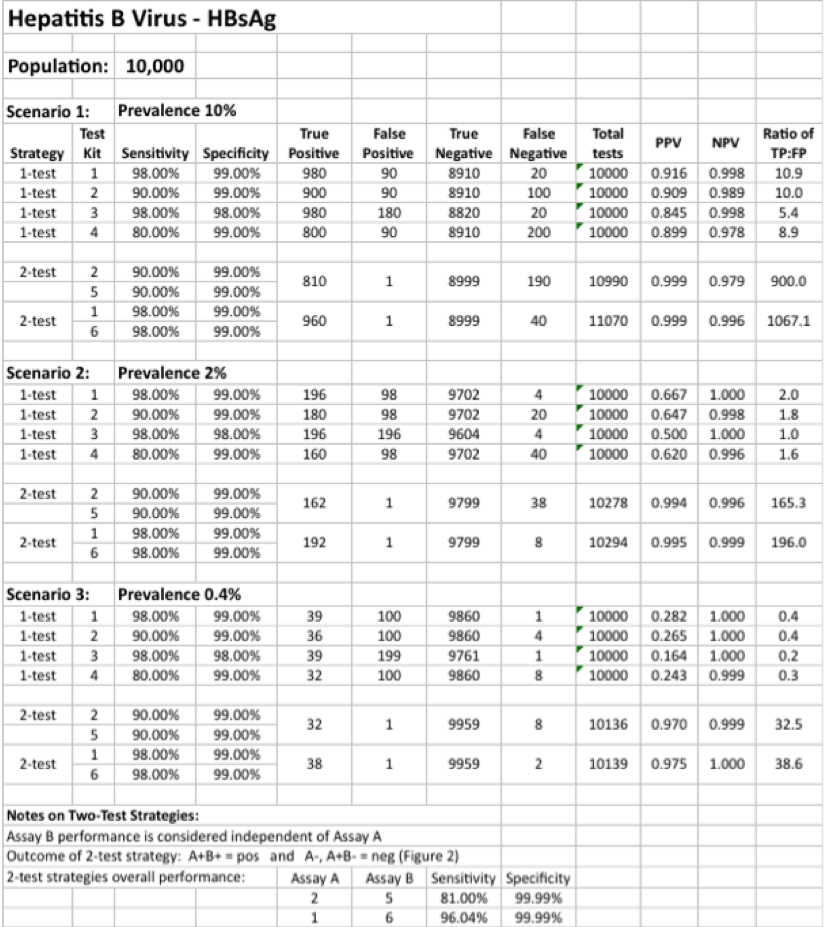

Supplement: Supplementary file 1 — Outcomes from applying 1- and 2-test diagnostic strategy models to three HBV epidemic scenarios. (DOCX 979 kb) [file 12879_2017_2774_MOESM1_ESM.docx]
